# Overexpression of a Cytosolic Abiotic Stress Responsive Universal Stress Protein (*Sb*USP) Mitigates Salt and Osmotic Stress in Transgenic Tobacco Plants

**DOI:** 10.3389/fpls.2016.00518

**Published:** 2016-04-21

**Authors:** Pushpika Udawat, Rajesh K. Jha, Dinkar Sinha, Avinash Mishra, Bhavanath Jha

**Affiliations:** ^1^Marine Biotechnology and Ecology Division, CSIR-Central Salt and Marine Chemicals Research InstituteBhavnagar, India; ^2^Academy of Scientific and Innovative Research, Council of Scientific and Industrial ResearchNew Delhi, India

**Keywords:** abiotic stress, drought, halophyte, salinity, transgenic

## Abstract

The universal stress protein (USP) is a ubiquitous protein and plays an indispensable role in plant abiotic stress tolerance. The genome of *Salicornia brachiata* contains two homologs of intron less *SbUSP* gene which encodes for salt and osmotic responsive USP. *In vivo* localization reveals that *Sb*USP is a membrane bound cytosolic protein. The role of the gene was functionally validated by developing transgenic tobacco and compared with control [wild-type (WT) and vector control (VC)] plants under different abiotic stress condition. Transgenic lines (T_1_) exhibited higher chlorophyll, relative water, proline, total sugar, reducing sugar, free amino acids, polyphenol contents, osmotic potential, membrane stability, and lower electrolyte leakage and lipid peroxidation (malondialdehyde content) under stress treatments than control (WT and VC) plants. Lower accumulation of H_2_O_2_ and $O_{2}^{-}$ radicals was also detected in transgenic lines compared to control plants under stress conditions. Present study confers that overexpression of the *SbUSP* gene enhances plant growth, alleviates ROS buildup, maintains ion homeostasis and improves the physiological status of the plant under salt and osmotic stresses. Principal component analysis exhibited a statistical distinction of plant response to salinity stress, and a significant response was observed for transgenic lines under stress, which provides stress endurance to the plant. A possible signaling role is proposed that some downstream genes may get activated by abiotic stress responsive cytosolic *Sb*USP, which leads to the protection of cell from oxidative damages. The study unveils that ectopic expression of the gene mitigates salt or osmotic stress by scavenging ROS and modulating the physiological process of the plant.

## Introduction

Abiotic stresses, especially salinity and drought are an important limiting factor in plant growth due to increased salinization of soil and water as well as the global scarcity of water resources (Vinocur and Altman, 2005). Abiotic stresses (salinity, dehydration, extreme temperature, highlight intensity, etc.) pessimistically impinge on plant growth and development through biomass accumulation and grain yield. Abiotic stresses are multigenic in nature, and the cellular responses are often interconnected. Thus, abiotic stress tolerance in a plant is synchronized action of multiple stress responsive genes, which communicate in stress signal transduction pathways with other components (Tuteja, 2007). Salinity negatively affects plant growth in two distinct phases; initial osmotic phase and the late ionic phase (Munns and Tester, 2008). These phases can be experimentally distinguished by measuring effects in the short term (within minutes to a few days) upon addition of salt or after longer (several days to weeks) times (Yamamoto et al., 2015). The cellular and molecular responses include accumulation of stress proteins, up regulation of stress responsive genes for antioxidants, transcription factors, compatible solutes, transporters/channels, and phytohormones, etc. Molecular mechanism of stress responses plays a key role in devising a strategy to engineer stress tolerant plants (Zahur et al., 2009). It is observed that abscisic acid (ABA) accumulates under drought stress condition, which promotes expression of numerous ABA dependent drought and abiotic stress responsive genes. Though substantial researches have been carried on the abiotic stress response of plants, there is inadequate information on molecular regulatory mechanisms of environmental stress responsive genes and proteins (Isokpehi et al., 2011). There are a number of reports on stress associated genes and proteins, but they are still functionally unknown or poorly characterized. Functional validation of these stress responsive genes or proteins will unveil a new regulatory molecular mechanism, which may consider a promising approach for developing crop plants, tolerant to environmental stresses.

The universal stress proteins (Usp) were named initially as C13.5 protein on the basis of their migration on two-dimensional IEF-PAGE gel. Nevertheless, UspA quickly became extensively acknowledged, and the protein has specified its name to a rising orthologous group of proteins, the UspA superfamily (Kvint et al., 2003). The universal stress protein A (UspA) superfamily covers primordial and preserved group of proteins originated in bacteria, archaea, fungi, flies, and plants. Kerk et al. (2003) reported enigmatic functions of USPA containing protein kinases in *Arabidopsis*. Even though the regulation of the *E*. *coli UspA* gene has been known to some extent, but the precise role of Usp proteins and Usp domains stay unfathomable, they have been emerged to be linked for providing resistance to DNA damaging agents and respiratory uncouplers. In *E. coli*, the USPs have been grouped into four classes according to their structural analysis and amino acid sequence; Class-I: UspA, UspC, UspD; Class-II: UspF and UspG; Class-III and Class-IV: Two Usp domains of UspE (Isokpehi et al., 2011). There are six families of *USP* genes *viz.* UspA, UspC, UspD, UspE, UspF, and UspG, whereas, UspB is not considered as a *bona fide* Usp family protein (Kvint et al., 2003). The six USP genes of *E. coli* have different functions linked to motility, adhesion and oxidative stress resistance (Loukehaich et al., 2012). It is observed that the majority of Usp genes are monocistronically expressed from σ^70^ promoter, but high temperature, ethanol, and tetracycline stress reduce the expression level of Usp proteins (Kvint et al., 2003). This might be due to the inactivation of σ^70^ promoter at high temperature stress. *USP* homologs are ubiquitous in plants and encoded by gene families while the functions of USPs remain largely unknown (Loukehaich et al., 2012). It appears that USP boost up the rate of cell survival during prolonged exposure to stress agents, and it may endow plants with wide ranging stress endurance activity (Zahur et al., 2009). The UspA protects cells from detrimental effect of toxic chemicals and osmotic stress or UV light (Kerk et al., 2003). Few plant *USP* genes have been characterized *viz. Gossypium arboreum*, *Astragalus sinicus*, *Vicia faba*, *Solanum pennellii*, Barley, and *Viridiplantae* (Sauter et al., 2002; Chou et al., 2007; Merkouropoulos et al., 2008; Maqbool et al., 2009; Zahur et al., 2009; Li et al., 2010; Isokpehi et al., 2011; Loukehaich et al., 2012). USPs are divided into two categories; first group binds with ATP (UspF and UspG-type) and second do not, such as UspAs and UspA-like group (Tkaczuk et al., 2013). The number of copies of *USPs* varies amongst different organisms and majority of them have many paralogous of *USPs*. For instance, the known copies of genes encoding *USPs* in *A. thaliana* are four, *Nitrosomonas europaea* and *Archaeoglobus fulgidus* are six and eight, respectively (Tkaczuk et al., 2013). Kvint et al. (2003) proposed that the ATP-binding Usp domain is a member of a group of α/β domains called the HUP domain (HIGH-signature proteins, *Usp*-like domains, and PP-ATPases).

A halophyte *Salicornia brachiata* requires NaCl for tissue culture (Joshi et al., 2012), owns sulfur rich seed storage proteins (Jha et al., 2012), unique oligosaccharides (Mishra et al., 2013), and metabolites (Mishra et al., 2015). Several candidate genes from this halophyte extensively explored for abiotic stress responsive genes and promoters for developing abiotic stress tolerant transgenic plants (Jha et al., 2011; Chaturvedi et al., 2012, 2014; Joshi et al., 2013; Singh et al., 2014a,b; Tiwari et al., 2014, 2015, 2016; Udawat et al., 2014). The present study addresses first report on the functional characterization of the *SbUSP* gene by ectopic expression in a model plant and further analyses under varying abiotic stress condition. It was observed that overexpression of the *SbUSP* gene mitigates salt and osmotic stress and provides endurance to the transgenic plant by modulating physiological processes.

## Materials and Methods

### Genomic Organization of *SbUSP* Gene

Plant (*S. brachiata*) genomic DNA was extracted using modified CTAB (*N*-cetyl-*N*,*N*,*N*-trimethyl ammonium bromide) method (Bubner et al., 2004) with minor modifications, quantified using ND-1000 spectrophotometer and qualitatively analyzed by agarose gel electrophoresis. The *SbUSP* gene was amplified with primer pairs (F: 5′-ATGGCAATGTCTGATAAACC-3′ and R: 5′-TCAGTGCTTAATCTTAGGCTT-3′) using genomic DNA as template, cloned into the pGEM-T easy vector and sequenced (M/s Macrogen, S. Korea).

The copy number of *SbUSP* gene was determined using Southern blot analysis. Genomic DNA (2 μg) was digested with restriction enzymes (*EcoR*1 and *BamH*1) and transferred to Hybond N^+^ membrane (Amersham Pharmacia, UK) using alkaline transfer buffer (0.4 N NaOH along with 1 M NaCl) by upward capillary transfer. The blot was hybridized overnight at 42°C with PCR generated DIG-11-dUTP labeled *SbUSP* probe (F: 5′-ATGGCAATGTCTGATAAACC-3′ and R: 5′-AGGAGGACAATGCGTGCTT-3′) and signals were detected on X-ray film by using CDP-Star as a chemiluminescent substrate, as described by manufacturer instructions (Roche, Germany).

### Subcellular Localization

The gateway technology was used to generate translational fusion cassette of *Sb*USP along with RFP (red fluorescent protein). The *SbUSP* CDS was PCR amplified using accuPrime *Pfx* DNA polymerase along with *SbUSP*F (5′-**CACC**ATGGCAGAAGTTGATT-3′) and *SbUSP*R (5′-TCAGTGCTTAATCTTAGGCTT-3′) primers. The PCR product, amplified with blunt ends was cloned into entry vector (pENTER/D-TOPO, Invitrogen, USA) and sequenced. Subsequently, LR recombination reaction was executed between an attL-containing entry clone pENTER/D-TOPO-*Sb*USP vector and an attR-containing destination vector pSITE-4CA (RFP) using gateway LR Clonase II enzyme mix (Invitrogen, USA). Cloning was confirmed by PCR, and resulting expression cassette (RFP:*Sb*USP) and control vector (pSITE-4CA:RFP) were transformed to onion epidermal cells using particle bombardment with gene gun (PDS-1000/He Biolistic, Bio-Rad, USA). Transformants were observed after incubation in dark for 12 h for transient expression of RFP using an epifluorescence microscope (Axio Imager, Carl Zeiss AG, Germany).

### Genetic Transformation to Tobacco Plants and Molecular Confirmation

The *SbUSP* CDS was amplified using proofreading enzyme accuPrime *Pfx* DNA polymerase along with primer pairs (F: 5′-**CTCGAG**ATGGCAATGTCTGATAAACC-3′ and R: 5′-**GGTACC**TCAGTGCTTAATCTTAGGCTT-3′) having *Kpn*I and *Xho*I sites, respectively. The *SbUSP* cDNA was cloned in pCAMBIA1301 *via* an intermediate pRT101 plant expression vector and recombinant pCAMBIA1301:35S:SbUSP vector was mobilized into *Agrobacterium tumefaciens* strain LBA4404. Tobacco (*Nicotiana tabacum* cv. Petit Havana) plants were transformed by leaf disk method (Horsch et al., 1985) and shoot buds directly generated from leaf disks were transferred to shoot elongation and subsequently to rooting media supplemented with hygromycin (20 mgL^-1^).

Putative transgenic lines (T_0_ and T_1_) were screened by growing on hygromycin (20 mgL^-1^), and the presence of transgenes was confirmed by PCR amplification of *uid*A (F: 5′-GATCGCGAAAACTGTGGAAT-3′ and R: 5′-TGAGCGTC GCAGAACATTAC-3′) and *SbUSP* genes. The activity of reporter gene β-glucuronidase in the leaf segment of transgenic (overexpressing *SbUSP* gene), VC (vector control; transgenic plant transformed with pCAMBIA1301 vector only) and WT (wild-type plant, non-transgenic) plants was visualized *via* GUS activity (Jefferson, 1987) using β-glucuronidase reporter gene staining kit (Sigma, USA). The integration of transgene and its copy number was determined by Southern blot analysis following manufacturer instructions (Roche, Germany).

### Overexpression Analysis of *SbUSP* Gene

Total RNA was extracted from transgenic, VC and WT plants (abiotic stressed and unstressed), quantified and cDNA was prepared using Superscript II RT (Invitrogen, USA). The semi-quantitative reverse transcriptase PCR (RT-PCR) was performed thrice independently (each containing three plants) for the *SbUSP* gene and β-tubulin gene (F: 5′-GGAGTCACCGAGGCAGAG-3′ and R: 5′-ATCACATATCAGAAACCACAA-3′) was used as an internal reference. Similarly, quantitative Real Time PCR (qRT-PCR) was performed to study the relative fold expression of *SbUSP* gene in transgenic lines under salinity stress.

### Analyses of T_1_ Transgenic Plants Exposed to Varying Abiotic Stresses

The T_1_ seeds (25 seeds from each plant) of *SbUSP* overexpressing transgenic lines (L7, L11, L14) along with VC and WT plants were germinated on MS media supplemented with NaCl (200 mM) or mannitol (300 mM) for three weeks under controlled conditions, and percentage seed germination was studied (Pandey et al., 2015). Percent germination was repeated three times and each contained three biological replicates. Equal size of hygromycin (20 mgL^-1^) positive T_1_ seedlings were transferred to MS media supplemented with NaCl (200 mM) or PEG (10%) and subsequently incubated for 21 days for root length, shoot length, and ion content analyses. Additionally, 45 days old plantlets (transgenic lines, VC and WT) were subjected to NaCl (200 mM), PEG (10%), ABA (20 μM) or SA (10 μM) stress for 24 h; thereafter samples were harvested. The function of *SbUSP* gene in transgenic plants was validated through morphological and physio-biochemical and molecular analyses *viz.* fresh weight, dry weight, osmotic potential, membrane stability index, electrolyte leakage, chlorophyll estimation, $O_{2}^{-}$ estimation, *in vivo* localization of H_2_O_2_ and $O_{2}^{-}$ radicals, etc.

### Quantification of Osmotic Adjustment

The leaf segments of 45 days old stressed and unstressed tobacco plants were harvested and incubated at -20°C. After overnight incubation, plant samples were thawed and centrifuged at 13,000 rpm for 10 min. The cell sap extract was used to measure cellular osmotic potential using vapor pressure osmometer (VAPRO, Wescor Inc., USA). The experiment was carried out three times, and each contained three biological replicates.

### Relative Water Content (RWC)

Fresh weight from control (WT and VC) and transgenic tobacco plants was determined, and leaf samples were floated overnight in deionised water. The turgid weight was determined, and all samples were dried at a constant temperature. Dry weight was measured, and percent relative water content (RWC, %) was calculated using following equation. RWC was measured three times, each containing three biological replicates.

$$RWC\;(\%)\,=100\,\times \frac{Fresh\,\;weight\,\;-Dry\,\;\;weight}{Turgid\,\;weight\,-Dry\,\;weight}$$

### Senescence Assay and Estimation of Chlorophyll Content

Leaf disks from 45 days old control and transgenic tobacco plants were punched out, and 11 leaf disks of similar size from each plant were floated in ½ MS (control) supplemented with NaCl (100, 150, or 200 mM) or PEG (10%) for 8 days. Effects of each treatment were gaged by visually analyzing phenotypic changes amongst leaf segments. Furthermore, total chlorophyll contents were isolated from leaf disks and determined as per gram fresh weight of tissue (Inskeep and Bloom, 1985). The experiment was repeated three times with three biological replicates each.

### Superoxide Estimation and Cell Viability Test

Production of superoxide radicals under NaCl (200 mM) or PEG (10%) stress was quantified using 2,3-bis-(2-methoxy-4-nitro-5-sulfophenyl)-2H-tetrazolium-5-carboxanilide (XTT) assay (Hema et al., 2014). Leaf segments (of equal size and weight) were incubated for 5 h in potassium phosphate buffer (20 mM, pH 6.0) containing 500 μM XTT and increase in absorbance was recorded at 470 nm.

Similarly, leaf segments (similar weight and size) of 45 days old stressed and unstressed plants were used for estimation of cell viability by 2,3,5-triphenyltetrazolium chloride (TTC) assay (Hema et al., 2014) in sodium phosphate buffer (pH 7.4). Leaf segments were thoroughly washed with sterile water and incubated in TTC solution at room temperature in the dark for 6 h. Samples were boiled in 5 ml of 2-methoxy ethanol till aridness to dig-out bound formazan. Subsequently, 5 ml of 2-methoxy ethanol was again added, and absorbance was recorded at 485 nm. Both experiments were carried out three times and each contained three biological replicates.

### Membrane Stability Index (MSI) and Electrolyte Leakage (EL)

Healthy young leaves were collected from stressed and unstressed WT and transgenic tobacco plants, and MSI was calculated by the method described by Sairam (1994). Leaf segments were placed in sterile water, and one set (*L*_1_) was incubated at 40°C for 30 min whereas other set (*L*_2_) was incubated at 100°C for 10 min. Electrical conductivity of samples from both sets was measured, and MSI was calculated using following formula:

$$MSI=100\times 1-\frac{L_{1}}{L_{2}}$$

For electrolyte leakage, leaf samples were initially washed with sterile water to eliminate surface stacked electrolytes. Healthy young leaf samples were incubated in deionized water in closed vials and kept overnight on a rotary shaker. Thereafter, electrical conductivity was measured (*L*_t_) using conductivity meter (SevenEasy, Mettler Toledo AG 8603, Switzerland). Subsequently, leaf samples were autoclaved at 120°C for 15 min, further, they were cooled to 25°C, and electrical conductivity (*L*_0_) was again determined (Lutts et al., 1996). The percent electrolyte leakage was calculated using following formula:

$$EL\,\left(\%\right)\,=100\,\times \frac{L_{t}}{L_{0}}$$

The MSI and EL experiments were repeated three times with three biological replicates each.

### Quantification of Proline, H_2_O_2_, and Lipid Peroxidation

The free proline content and H_2_O_2_ level in the stressed and unstressed WT and transgenic tobacco plants were determined using methods described by Bates et al. (1973) and He et al. (2005), respectively. A standard curve was prepared using known amount of proline and H_2_O_2_, and absorbance was measured at 520 and 560 nm for determining free proline and H_2_O_2_ content, respectively. Lipid peroxidation was analyzed by quantifying the amount of MDA (malondialdehyde) generated by TBA (thiobarbituric acid) reaction (Draper and Hadley, 1990). All experiments were repeated three times with three biological replicates each.

### Quantification of Total Soluble Sugar, Reducing Sugar, Starch, Total Amino Acid, and Polyphenol Content

The total soluble sugar, reducing sugar and starch contents were determined against a standard curve prepared with glucose (Sigma–Aldrich, USA). Stressed and unstressed control and transgenic leaf samples were homogenized in 85% ethanol and treated with anthrone reagent. Samples were boiled, cooled up to 25°C, absorbance was measured at 620 nm and total soluble sugar was calculated from the standard curve. Reducing sugar was quantified by a colorimetric test using DNS method (Yemm and Willis, 1954). An aliquot of glucose or leaf sample was mixed with 3 ml of DNS reagent, boiled, cooled to room temperature, and color intensity was measured at 540 nm. For starch estimation, homogenized samples were treated with 52% perchloric acid, centrifuged, the supernatant was treated with 4 ml of anthrone reagent and absorbance was measured at 630 nm (Ghose, 1987). Free amino acids and polyphenol contents were determined against a standard curve prepared with glycine and catechol solution, respectively. The ethanolic extract of samples was redissolved in ninhydrin reagent or folin–ciocalteu reagent, and absorbance (color intensity) was measured at 570 or 650 nm, respectively. All experiments were carried out three times with three biological replicates each.

### *In Vivo* Localization of H_2_O_2_ and $O_{2}^{-}$

Histochemical staining with 3,3-diaminobenzidine (DAB) or nitro-blue tetrazolium (NBT) was performed for *in vivo* analyses of H_2_O_2_ and $O_{2}^{-}$ radical production (Shi et al., 2010). A measure of 1 mg ml^-1^ each stain solution was prepared in phosphate buffer (10 mM pH 7.8 for NBT and pH 7.3 for DAB). Leaf samples were immersed in the respective staining solution in the dark for 2 h, thereafter exposed to white light until blue and brown spots appeared. Total chlorophyll content was bleached and leaf samples were documented.

### Analyses of Ion Content

Seedlings from stressed and unstressed, control and transgenic plants were subjected to dryness at 70°C for 48 h and dry weight was determined. Samples were acid digested overnight in perchloric acid-nitric acid solution (3:1) heated to dryness and redissolved in sterile water. Ion content was measured using inductively coupled plasma optical emission spectrometer (Optima2000DV, PerkinElmer, Germany). ICP analysis was done three times with three biological replicates each.

### Quantitative RT PCR Analysis

The cDNA of all control and treated plant samples (WT, VC, and transgenic lines) *viz.* NaCl, PEG, ABA, and SA was synthesized from 5 μg total RNA using Superscript RT III first strand cDNA synthesis kit (Invitrogen, USA). The resulting cDNAs were subjected to real time qRT PCR analyses for selected antioxidant enzyme encoding genes of host plant (*NtSOD*, F: 5′-AGCTACATGACGCCATTTCC-3′ and R: 5′-CCCTGTAAAGCAGCACCTTC-3′; *NtAPX*, F: 5′-CAAATGTAAGAGGAAACTCAGAGGA-3′ and R: 5′-CAGCCTTGAGCCTCATGGTACCG-3′; *NtCAT*, F: 5′-AGGTACCGCTCATTCACACC-3′ and R: 5′-AAGCAAGCTTTTGACCCAGA-3′) followed by melt curve analysis for the validation of specificity of reaction. The relative fold expression change was calculated using the CT method, and internal reference gene β-tubulin was used as a reference (Livak and Schmittgen, 2001).

### Statistical Analysis

For each set of experiment, data from three replicates, each containing three biological replicates were documented. Data sets were articulated as mean ± SE and were used to determine the significance of difference by analysis of variance (ANOVA) amongst the means of WT, VC, and transgenic plants of every treatment set. For comparison of multiple means Tukey HSD was used, *p* < 0.05 was considered significantly different from each other and designated by different letters. All dataset was analyzed individually and in combination by principal component analysis (PCA) and respective heat maps were generated. PCA is a multivariate analysis method by which correlation between variables were studied among multidimensional datasets. Plants grown under varying stress were selected as observations, whereas different morphological and physio-biochemical measurements were taken as variables. Observation and variable data set were used to generate Pearson’s correlation matrix, and PCA was analyzed using different software SigmaPlot (version 12), SYSTAT (version 13) and Origin (version 15).

## Results

### *In Vivo* Localization and Genome Organization

**FIGURE 1:**
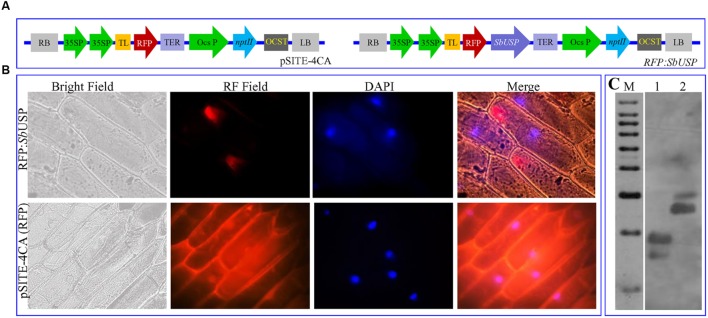
**Sub-cellular localization of *Sb*USP protein and genome organization. (A)** Vector *pSITE-4CA*(RFP) and *SbUSP-1:RFP* gene construct, **(B)** Transient expression of RFP alone and *Sb*USP:RFP translational fusion protein on transformed onion epidermal cells and **(C)** Southern hybridization of *Salicornia brachiata* genome to determine the copy number of *SbUSP* gene.

The *in vivo* subcellular localization study was accomplished by transient expression analysis of translational fusion construct pSITE-4CA:*Sb*USP and vector pSITE-4CA:RFP (**Figures 1A,B**) expressing RFP only in onion epidermal cells. The expression with pSITE-4CA vector showed uniform red fluorescence, whereas RFP:*Sb*USP fusion construct revealed fluorescence localized to the cytosolic membrane (**Figure 1B**). DAPI (4′,6-diamidino-2-phenylindole) is a fluorescent stain, which binds to double strand DNA at A-T rich region. DAPI has an emission maximum at 461 nm, and in fluorescence microscopy, it is detected through a blue filter. In the *in vivo* localization study, blue spot of DAPI indicates the position of the nucleus (as it binds with dsDNA). Overlapping of DAPI blue spots was not observed with RFP spots in the cells, transformed with RFP:*Sb*USP fusion construct. In the merged image, *Sb*USP bound RFP shows red fluorescence in the cytosol, outside the nucleus and inner side of the membrane. The result suggested that *Sb*USP is a membrane bound cytosol-localized protein. The *in silico* analysis also confirmed that *Sb*USP is membrane bound cytosolic protein. The *SbUSP* ORF amplified using full length gene specific primers from cDNA, and genomic DNA showed similar size, 486 bp. The comparative sequence analysis of cDNA clone compared to genomic clone further reveals single exon structure and confirms intron-less genomic organization the gene (data not shown). Furthermore, Southern blot analysis was commenced to identify the number of *SbUSP* copies (homologs) in the genome of *S. brachiata*. The analysis revealed a double copy of the *SbUSP* gene as the probe hybridized with two fragments of genomic DNA (**Figure 1C**).

### Molecular Analyses of Transgenic Tobacco Plants

**FIGURE 2:**
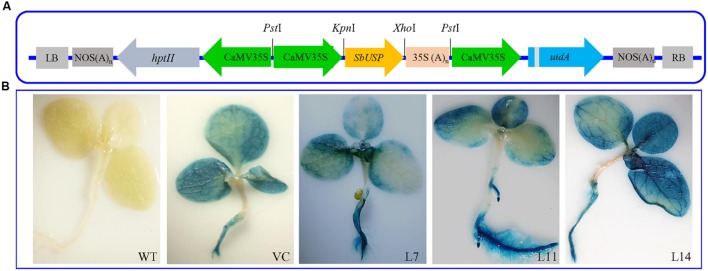
**Genetic transformation and Histochemical GUS assay of transgenic tobacco plants. (A)** Schematic representation of *SbUSP-pCAMBIA1301* plant transformation vector construct and **(B)** Histochemical GUS assay of transgenic lines (L7, L11, and L14) along with control plants (WT and VC).

Healthy leaves from the *SbUSP* gene (**Figure 2A**) overexpressing T_1_ lines (L1 to L17 lines), WT and VC plants were harvested and screened for the presence of transgenes (Supplementary Figure S1). Furthermore, Southern blot analysis confirmed presence of single copy transgene amongst L4, L7, L8, L11, L12, and L14 transgenic lines (Supplementary Figure S2A). The *SbUSP* gene overexpressing transgenic lines exhibited a high level of *gus* expression along with VC plants (**Figure 2B**). Comparative ectopic expression of the *SbUSP* gene was observed by semi-quantitative RT-PCR and quantitative Real Time PCR (qRT-PCR) for transgenic lines (Supplementary Figure S2) using β-tubulin as an internal reference gene. The analysis revealed high expression of the *SbUSP* gene in transgenic lines L7, L11, and L14, therefore, selected for further functional validation by morphological, physio-biochemical and molecular analyses.

### Overexpression of the *SbUSP* Gene Enhances Plant Growth under Salt and Osmotic Stress

**FIGURE 3:**
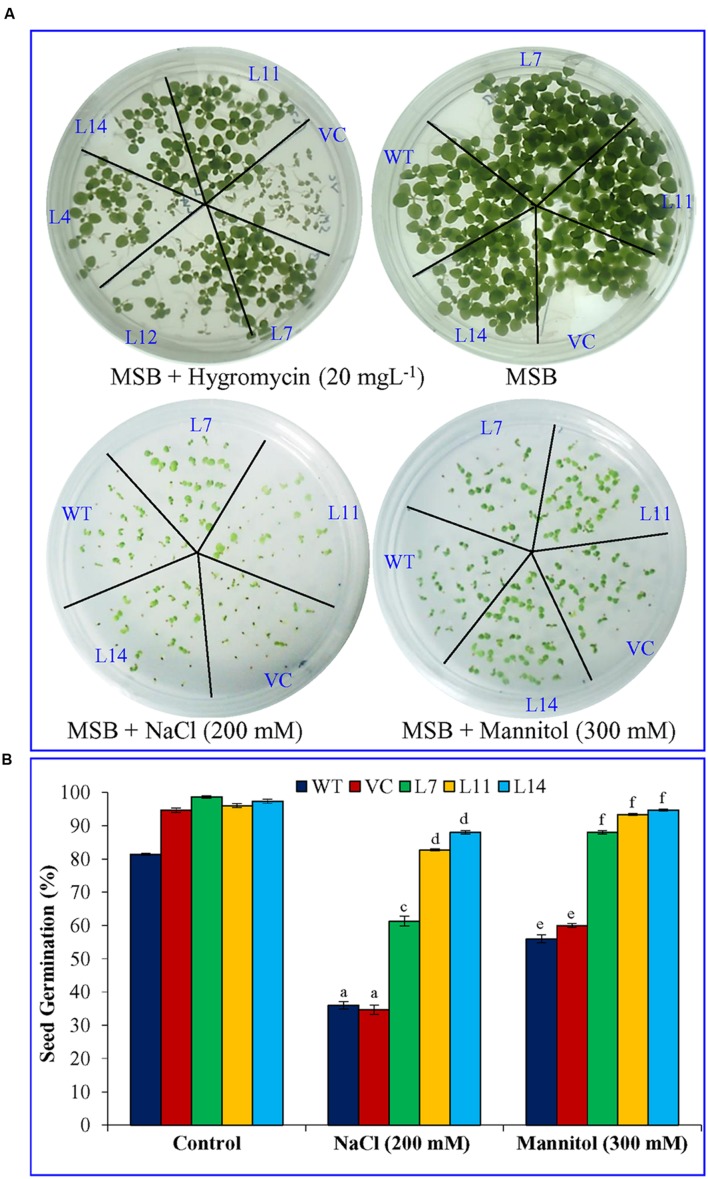
**Seed germination analysis of transgenic tobacco plants under abiotic stress. (A)** Morphology and **(B)** percent seed germination analysis of transgenic lines (L7, L11, and L14), WT and VC plants under salinity and osmotic stress condition. Bars represent means ± SE and values with different letters are significant at *P* < 0.05.

**FIGURE 4:**
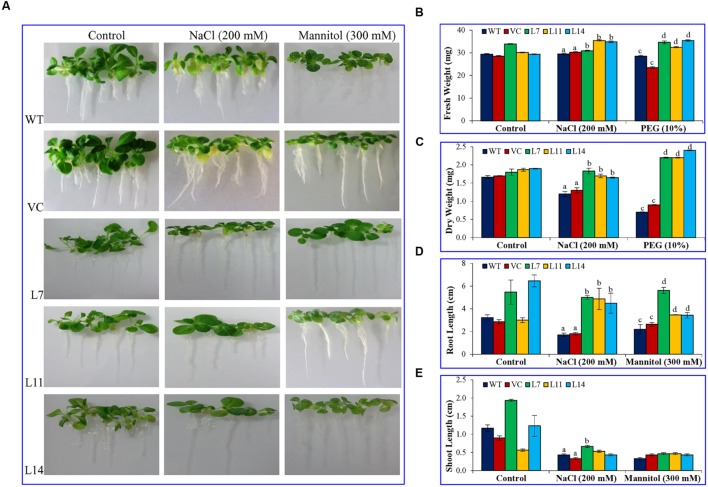
**Plant growth analyses under salt and osmotic stress.** Comparative study of **(A)** morphology, **(B)** fresh weight, **(C)** dry weight, **(D)** root length, and **(E)** shoot length of transgenic lines (L7, L11, and L14) and control plants (WT and VC) under salt and osmotic stress. Bars represent means ± SE and values with different letters are significant at *P* < 0.05.

**FIGURE 5:**
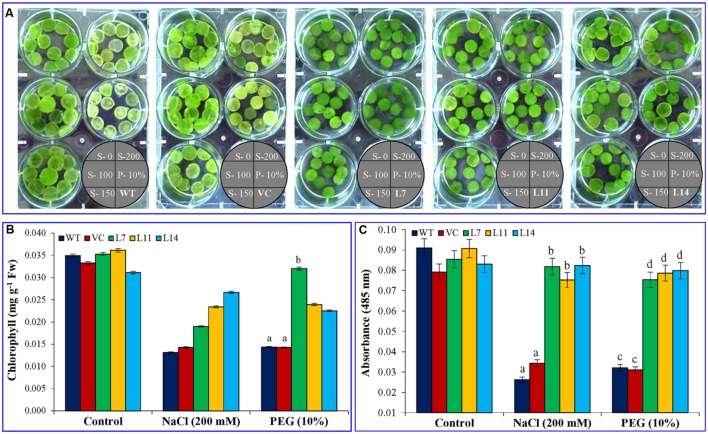
**Leaf disk assay, estimation of chlorophyll content and cell viability. (A)** Leaf disk assay, **(B)** total chlorophyll content, and **(C)** cell viability of transgenic (L7, L11, and L14) and control plants (WT and VC) under salt and osmotic stress condition. Bars represent means ± SE and values with different letters are significant at *P* < 0.05.

Percentage seed germination was determined under control, NaCl, and osmotic stress condition (**Figure 3**). It was observed that transgenic lines (L7, L11, and L14) showed significantly (*p* < 0.05) higher percent seed germination compared to control plants (WT and VC) under the stressed condition. Similarly, growth parameters, including fresh weight, dry weight, shoot and root length were significantly (*p* < 0.05) improved in selected transgenic lines (L7, L11, and L14) compared to control plants (WT and VC) under stress conditions (**Figure 4**). Leaf segments reduce their viability and undergo senescence under salt and osmotic stress (**Figure 5A**). Salt and osmotic stress induced damages were lower in *SbUSP* overexpressing transgenic lines compared to their counterparts (WT and VC). Transgenic lines exhibited reduced rate of necrosis and therefore, significantly (*p* < 0.05) higher amount of chlorophyll content was observed in transgenic plants related to WT and VC plants. A degradation in cellular chlorophyll content was observed in WT and VC plants compared to transgenic lines (**Figure 5B**). Under control condition, chlorophyll content of WT, VC, and transgenic plants was comparable, whereas under increasing salt and osmotic stress, significantly higher amount of chlorophyll was found in transgenic lines. Additionally, transgenic lines showed significantly (*p* < 0.05) enhanced cell viability compared to WT and VC plants under stress condition (**Figure 5C**).

### *SbUSP* Overexpression Alleviates ROS Buildup under Salinity and Oxidative Stress

**FIGURE 6:**
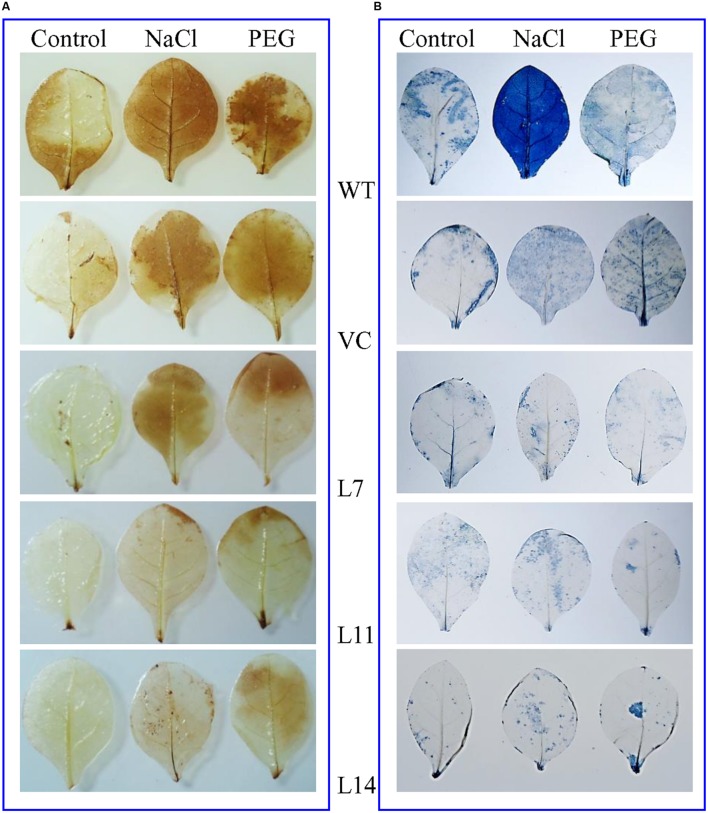
***In vivo* localization of peroxide and superoxide free radicals. (A)** DAB and **(B)** NBT staining of transgenic (L7, L11, and L14) and control (WT and VC) leaves.

The WT and VC plant leaves exhibited deeper NBT and DAB staining than transgenic lines exposed to salt and osmotic stress (**Figure 6**). Results confirmed that control plants (WT and VC) accumulate more $O_{2}^{-}$ and H_2_O_2_ compared to transgenic plants and thus validating the role of *SbUSP* gene in providing oxidative stress tolerance. Abiotic stress also leads to the generation of superoxide radicals. It was demonstrated by the reduction of tetrazolium salts XTT, resulting significant (*p* < 0.05) increase in absorbance (470 nm) in transgenic lines compared to control plants (WT and VC) under stress condition (Supplementary Figure S3).

**FIGURE 7:**
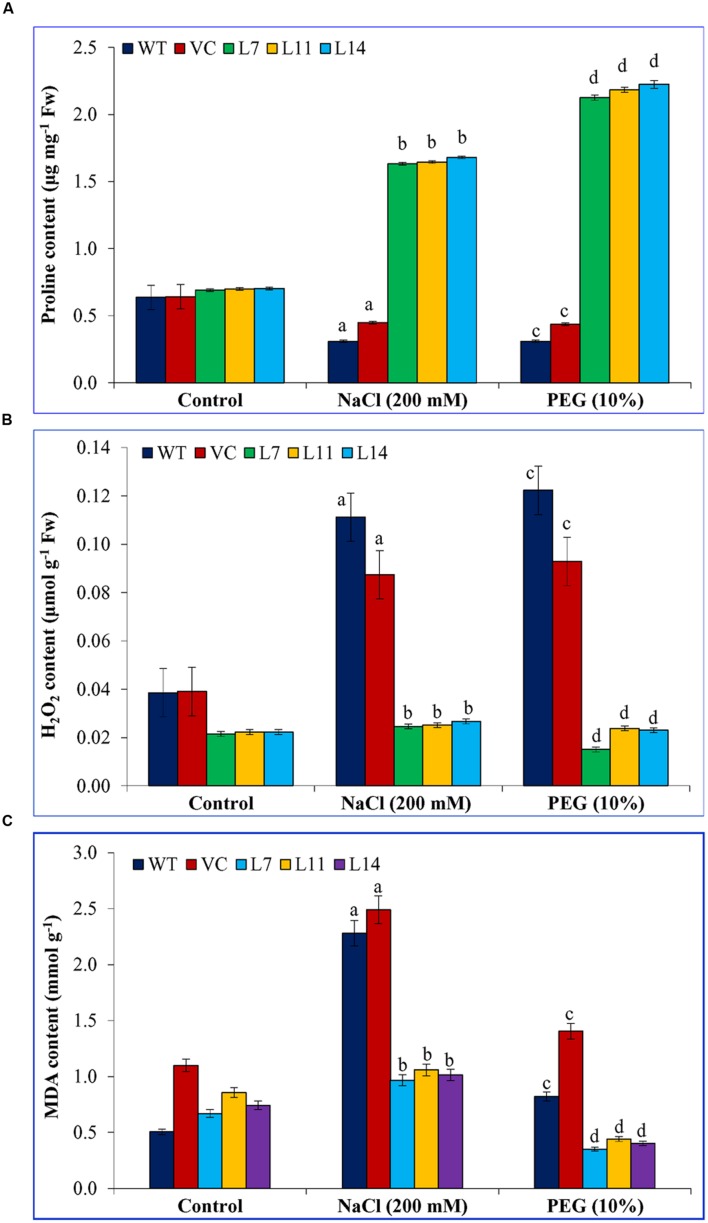
**Biochemical analyses of transgenic tobacco plants under abiotic stress.** Estimation of **(A)** proline, **(B)** H_2_O_2_, and **(C)** MDA contents in control (WT and VC) and transgenic plants (L7, L11, and L14) under salinity and osmotic stress condition. Bars represent means ± SE and values with different letters are significant at *P* < 0.05.

Proline and MDA contents were comparable in WT, VC, and transgenic lines under control condition (**Figure 7**). Transgenic lines accumulated significantly (*p* < 0.05) high proline compared to control plants under stress (**Figure 7A**). In contrast, elevated H_2_O_2_ and MDA contents (*p* < 0.05) were found in control plants under stress condition compared to transgenic lines (**Figures 7B,C**). Results further confirmed its (*SbUSP*) role in oxidative stress tolerance.

### Overexpression of the *SbUSP* Gene Improves Physiological Status of Plant under Stress

**FIGURE 8:**
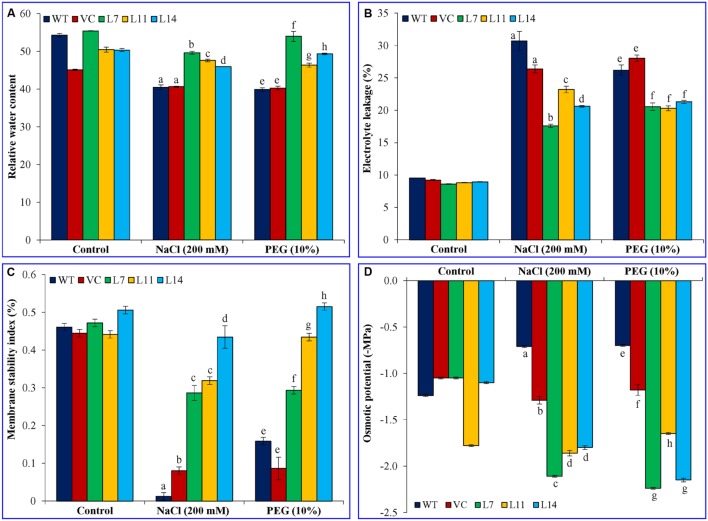
**Physiological analyses of transgenic lines.** Estimation of **(A)** RWC, **(B)** electrolyte leakage, **(C)** membrane stability index, and **(D)** osmotic potential from leaves of control plants (WT and VC) and transgenic lines (L7, L11, and L14) under control, salinity and osmotic stress conditions. Bars represent means ± SE and values with different letters are significant at *P* < 0.05.

Transgenic, WT and VC plants showed alike physiological status regarding RWC, EL, MSI, and OP under control condition (**Figure 8**). However, transgenic lines showed significantly (*p* < 0.05) higher RWC and MSI under stress condition compared to control plants. Furthermore, transgenic plants exhibited considerably (*p* < 0.05) reduced electrolyte leakage and osmotic potential under stress condition. Transgenic lines exhibited better osmotic adjustment and thus physiological status of transgenic plants improves.

### Total Soluble Sugar, Reducing Sugar, Free Amino Acids, and Polyphenol Contents Increase in the *SbUSP* Overexpressing Lines

**FIGURE 9:**
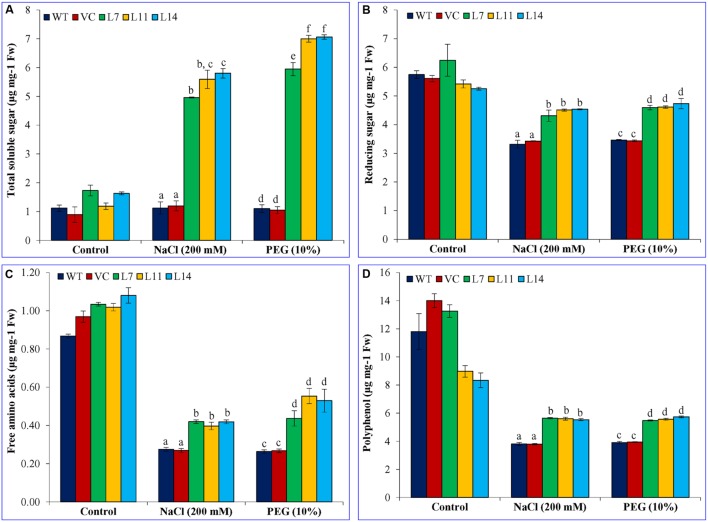
**Estimation of sugar, free amino acids, and polyphenol contents. (A)** Total soluble sugar, **(B)** reducing sugar, **(C)** free amino acids, and **(D)** polyphenol of transgenic lines (L7, L11, and L14) and control plants (WT and VC) under control and abiotic stress conditions. Bars represent means ± SE and values with different letters are significant at *P* < 0.05.

Total soluble sugar and reducing sugar were found almost similar in WT, VC, and transgenic lines under control conditions (**Figure 9**). A significant difference (*p* < 0.05) was observed amongst WT, VC, and transgenic plants exposed to salt and osmotic stress. Stress had no effect on total soluble sugar content in WT and VC plants, whereas transgenic lines accumulate significantly (*p* < 0.05) high content compared to control plants as well as the control condition (**Figure 9A**). The transgenic line L7 showed fivefold, whereas L11 and L14 showed a sixfold increase of soluble sugar content under salt stress. Similarly, under osmotic stress, line L7 showed more than sixfold increase and line L11 and L14 showed eightfold increases in total soluble sugar content. Reducing sugar, free amino acid and polyphenol content decreased under stress condition compared to the control condition, irrespective of plants. But, transgenic lines accumulated significantly (*p* < 0.05) higher content of reducing sugar, free amino acid and polyphenol compared to control plants (WT and VC) under stress condition (**Figures 9B–D**). Results showed the accumulation of primary metabolites/osmolytes in transgenic plants, which improve the performance of transgenic plants under stress condition.

### Overexpression of the *SbUSP* Maintains Ion Homeostasis

For analyses of ion content, the WT, VC, and *SbUSP* overexpressing transgenic seedlings were grown on MS media supplemented with NaCl (200 mM) and grown for 28 days. The Na^+^ and K^+^ content in whole seedlings were measured in WT, VC, and transgenic seedlings exposed to salt stress. Potassium ion content was increased in transgenic lines compared to control plants and control conditions (Supplementary Figure S4). In contrast, Na^+^ accumulation was observed in control plants (WT and VC) compared to transgenic lines under salt stress. The K^+^/Na^+^ ratio, an indicator of ion homeostasis was almost similar in all plants under control condition. The ratio decreased in control plants (WT and VC) under salt stress, whereas elevated ratio was detected in transgenic lines compared to control plants and control condition (Supplementary Figure S4). The high K^+^/Na^+^ ratio indicates that transgenic lines improved their ion homeostasis under salinity and thus performed better and showed stress endurance.

### The *SbUSP* Gene May Also Involve in Transcriptional Regulation of Antioxidant Enzyme-Encoding Genes

**FIGURE 10:**
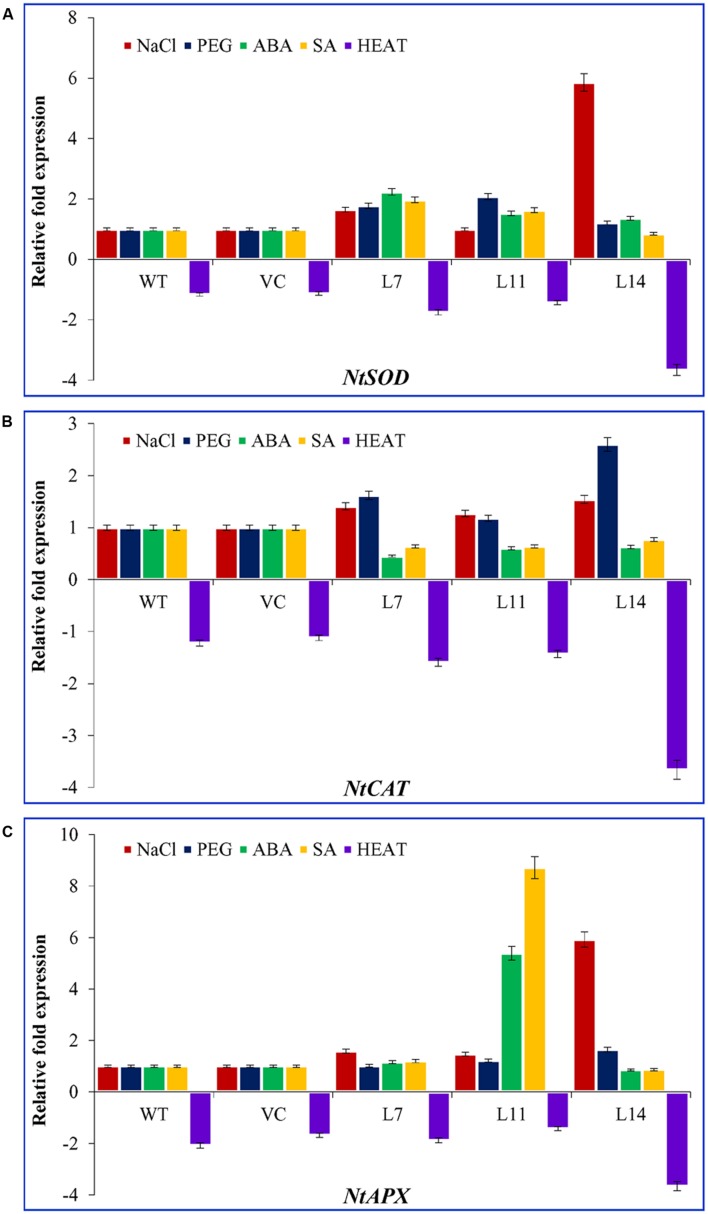
**Transcript expression analysis of three antioxidant enzymes encoding genes.** Comparative expression profile of **(A)** *NtSOD*, **(B)** *NtCAT*, and **(C)** *NtAPX* genes (encoding for superoxide dismutase, catalase, and ascorbate peroxidases enzymes, respectively) of transgenic lines (L7, L11, and L14) and control plants (WT and VC) under abiotic stress (salinity, osmotic, ABA, SA, and heat) conditions. Bars represent means ± SE and values with different letters are significant at *P* < 0.05.

Transcript expression of ROS scavenging enzyme genes *viz.* superoxide dismutase (*NtSOD*), catalase (*NtCAT*), and ascorbate peroxidase (*NtAPX*) were studied in transgenic lines under different abiotic stress condition, such as salt (200 mM), PEG (10%), heat (45°C), ABA (20 μM) and SA (10 μM), and compared with control plants and control condition (**Figure 10**). The transcript of *NtSOD*, *NtCAT*, and *NtAPX* genes was nearly steady in control plants (WT and VC) under every stress treatment compared to transgenic lines. A heat map showed the differential expression of antioxidant genes under varying stress condition (Supplementary Figure S5). The expression of *NtSOD* transcript under NaCl stress was increased up to twofold and sixfold in L7 and L14, respectively. Similarly, under osmotic stress, the transcript of lines L7 and L11 was up regulated by twofold. Exogenous application of ABA and SA increased the expression level of *NtSOD* transcript in L7 and L11. In addition, the expression of *NtCAT* was increased up to twofold and threefold in L7 and L14 under osmotic stress. Likewise, under salt stress, *NtCAT* transcript was accumulated up to twofold, whereas exogenous application of ABA and SA reduce the expression of *NtCAT* transcript. In contrast, ABA and SA treatments increased the expression of *NtAPX* transcript up to sixfold and ninefold, respectively, in L11. Similarly, the transcript of *NtAPX* accumulated by twofold and sevenfold in L7, L11, and L14 under salt stress. No significant difference was observed in *NtAPX* transcript level under osmotic stress. The transcripts of *NtSOD*, *NtCAT*, and *NtAPX* were down regulated under heat stress in all plants (WT, VC, and transgenic). However, down-regulation was significantly higher in transgenic lines compared to control plants. Transcriptional regulation of antioxidative genes in *SbUSP* overexpressing transgenic lines explicated its probable role as signaling molecule in salt and osmotic stress tolerance.

### Multivariate Data Analysis Distinguished Plant Responses under Stress Condition

**FIGURE 11:**
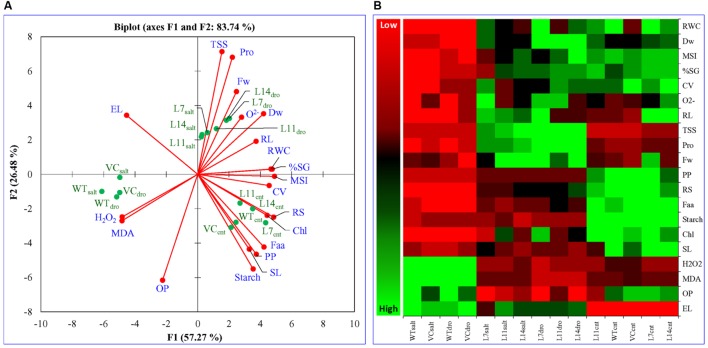
**Multivariate data analysis.** An integrated comparative **(A)** Bi-plot based principal component analysis (PCA) with first two principal components and **(B)** heat map showing the differential response of transgenic lines (L7, L11, and L14) and control plants (WT and VC) under normal and stress (NaCl and osmotic) condition. PCA is a multivariate analysis method by which correlation between variables was studied among multidimensional datasets. Plants grown under varying stress were selected as observations, whereas different morphological and physio-biochemical measurements were taken as variables. Observation and variable data set were used to generate Pearson’s correlation matrix, and PCA was analyzed.

**FIGURE 12:**
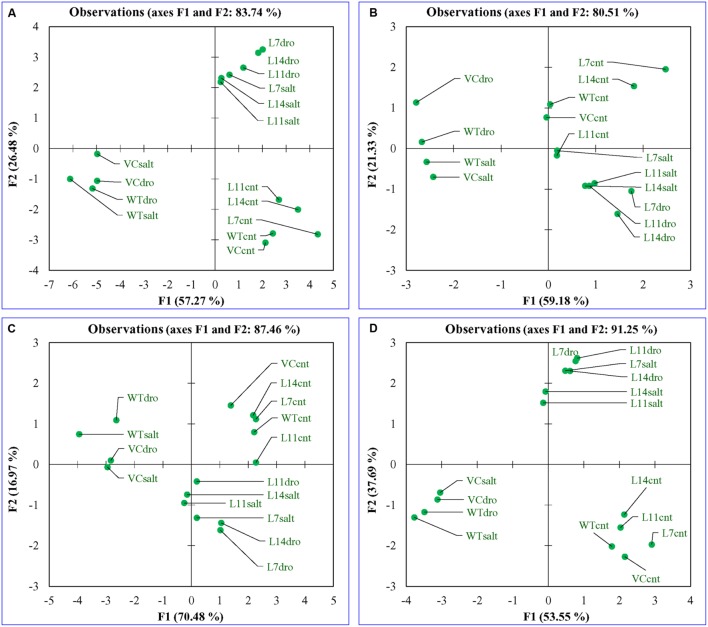
**Principal component analysis.** Comparative PCA of **(A)** combine, **(B)** morphological, **(C)** biochemical, and **(D)** physiological response of transgenic lines (L7, L11, and L14) and control plants (WT and VC) under control and stress (NaCl and osmotic) condition. Individually, the morphological, biochemical, and physiological response of plants explained 80.51, 87.46, and 91.25% of variations, respectively, whereas in-combination they explained 83.74% variations.

Principal component analysis was performed for the comparative study of the morphological, biochemical, and physiological response of transgenic lines (L7, L11, and L14) and control plants (WT and VC) under normal and different stress (NaCl and osmotic) conditions. The integrated PCA showed the possible correlation of plant response to different variables and stress condition (**Figure 11**). Individually, the morphological, biochemical, and physiological response of plants explained 80.51, 87.46, and 91.25% of variations, respectively (**Figure 12**), whereas in-combination they explained 83.74% variations because of varying response as shown by heat map (**Figure 11**).

**FIGURE 13:**
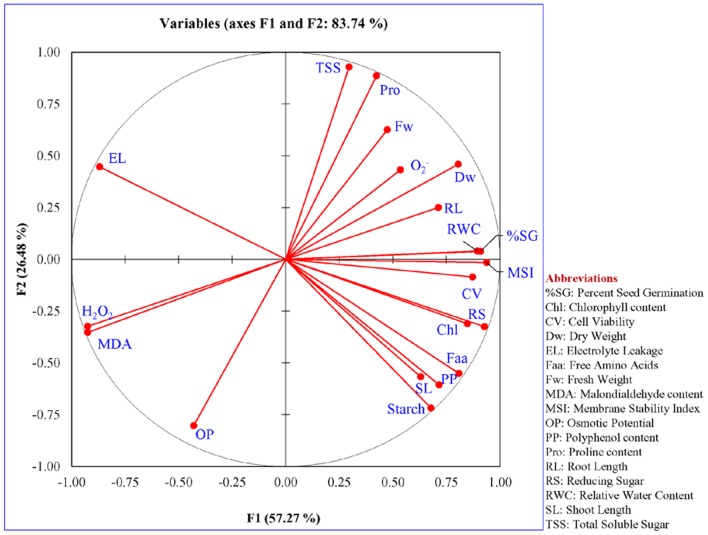
**Statistical significant analysis of variables.** The significance of parameters used to study the response of transgenic (L7, L11, and L14) and control (WT and VC) plants under different abiotic stress condition. A notable response was observed in the integrated PC analysis, showing a significant correlation of plant response and the stress condition.

All plants (transgenic lines and control) showed comparable response under un-stress condition as revealed by the bi-plot analysis of individual and integrative analysis (**Figures 11** and **12**). Control plants (WT and VC) clustered together in all analysis (individual and integrated) and exhibited a similar response toward stress (salinity or drought) condition. Similarly, under stress condition, transgenic lines assembled at an axis and unveiled alike response to combat with environmental stress. A remarkable response was observed in the integrated PC analysis, which showed a significant correlation of plant response and the stress condition (**Figure 13**). Overall, the PCA exhibited a statistical distinction among morphological and physio-biochemical responses of control plants (WT and VC) and transgenic plants, also under control and stress conditions (salinity and drought).

## Discussion

Due to sessile nature, plants have to widen their adaptive strategies to survive against continuous exposure to biotic and abiotic stresses. In a study of isolation and characterization of novel salt responsive genes, a novel gene encoding USP A-like from a euhalophyte *S. brachiata* (*SbUSP* A-like) has been isolated and functionally validated. The *SbUSP* gene encodes a protein that contains conserved 140–160 residues of a USP domain (Pfam Accession: PF00582) and has the ability to respond to a plethora of environmental stresses in bacteria, archaea, fungi, protozoa, and plants (Isokpehi et al., 2011). Some proteins of USP family in archaea, cyanobacteria, and plants are large proteins in which, the Usp domain is present together with other functional domains like Na^+^/H^+^ antiporter domains, Cl^-^ voltage channels, amino acid permeases and protein kinase domains (Kvint et al., 2003). The present study explored the function of the *SbUSP* gene under different abiotic stresses in a model plant tobacco. In plants, very few homologs of the USP family have been isolated and characterized (Sauter et al., 2002; Chou et al., 2007; Merkouropoulos et al., 2008; Maqbool et al., 2009; Zahur et al., 2009; Li et al., 2010; Isokpehi et al., 2011; Loukehaich et al., 2012). The structure alignment and *in silico* analyses showed phosphorylation, glycosylation, and ATP binding sites (84% probability) of *Sb*USP and its interaction with adenosine monophosphate (Udawat et al., 2014). The *Sb*USP is a phosphoprotein that contains autophosphorylating serine and threonine residue and uses either GTP or ATP as phosphate donors (Kvint et al., 2003; Udawat et al., 2014). The multiple amino acid sequence alignment followed by phylogenetic analyses revealed that *Sb*USP clustered with *Rc*USP and generated a separate clad distinct from the USP of other plant species (Udawat et al., 2014). Based on pattern of conserved structural features, it was proposed that the USPA domain is a part of larger structural families, whose members has already diversified and present in the last universal common ancestor of all extant of life, furthermore it was suggested that ancestral function of the USPA domain was nucleotide binding and signal transduction (Kerk et al., 2003).

The Southern blot analysis identified two homologs of *SbUSP* in *S. brachiata* genome and the genome organization study revealed intronless genomic arrangement of the gene *SbUSP* (**Figure 1**). Subcellular localization, determined using *35S::SbUSP::RFP* translational fusion construct, showed red fluorescence exclusively in the cytosol (**Figure 1**) and it is evident that *Sb*USP phosphoprotein is present in the cytosol as membrane bound form. The *in silico* study also predicted that *Sb*USP is membrane bound cytosolic protein (Udawat et al., 2014). Loukehaich et al. (2012) determined the subcellular localization of *SpUSP* using *35S::SpUSP::GFP* translational fusion construct and detected green fluorescence exclusively in nucleus and cell membrane, whereas cells transformed with the vector containing GFP alone displayed fluorescence throughout the cells (**Figure 1**). To understand the role of *SbUSP* gene in abiotic stress tolerance, three homozygous transgenic tobacco lines (T_1_) L7, L11, and L14, containing single gene integration, overexpress high transgene(s) transcripts (Supplementary Figure S2) and high seed germination percentage under stress condition (**Figure 3**) were selected.

**FIGURE 14:**
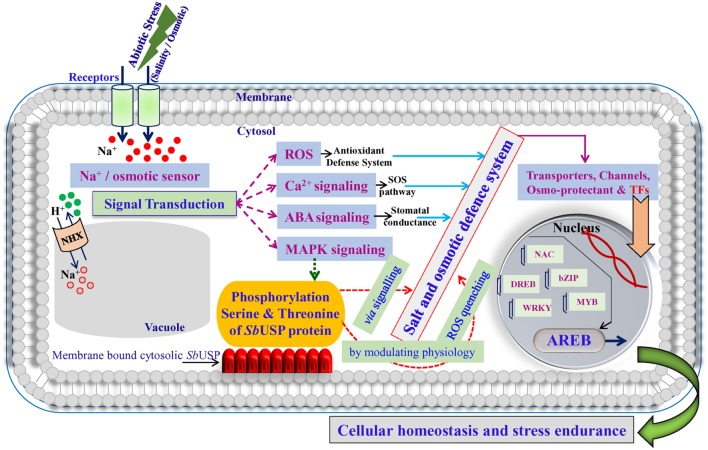
**A hypothetical model for the role of membrane bound *Sb*USP in abiotic stress defense system**.

Transgenic lines showed improved plant biomass including, root length, shoot length, fresh weight (FW), and dry weight (DW) under different stress conditions compared to control plants (**Figure 4**), which reveal that the *SbUSP* gene reduces deleterious effects of salt and osmotic stresses. It is predicted that overexpression of *SbUSP* alters the development of the plant probably by changes in levels of phytohormones, like auxin and cytokinin. Furthermore, enhanced vegetative growth was observed in transgenic lines even under normal conditions, which is supported by the comparable chlorophyll content of transgenic lines (**Figure 5**). It is speculated that *SbUSP* gene regulates the expression of antioxidant encoding genes (**Figure 10**) and also induces genes of the abiotic stress defense system (**Figure 14**), which in turn provide stress endurance. It was established that total chlorophyll content, one of the markers of cellular stress decreased significantly under stress condition because of ROS generation in the chloroplast (Bartels and Sunkar, 2005). ROS inhibits the PSII repair system, which resulted in the degradation of chlorophyll (Bartels and Sunkar, 2005). In the present study, transgenic plants exhibited significantly higher chlorophyll content and also reduced leaf senescence under oxidative stress (**Figure 5**). Based on results, it is presumed that the *SbUSP* gene may be involved in the mechanism that protects chlorophyll from oxidative damage. Plants grown under control condition did not show the generation of peroxide and superoxide free radicals in a localization study (**Figure 6**). Control plants (WT and VC) showed brown and blue-colored precipitate after DAB and NBT staining, which indicates a higher level of free radicals compared to transgenic lines under stress condition. Similarly, negligible H_2_O_2_ content was measured in transgenic lines compared to control plants (WT and VC), which showed significant (*p* < 0.05) accumulation of H_2_O_2_ content under stress condition (**Figure 7B**). These observations indicate the role of *SbUSP* gene in alleviating oxidative stress by ROS scavenging activity. Recently, USP mediated ROS homeostasis was reported under anoxic conditions and oxygen sensing ability of USP followed by ROS signaling is proposed (Gonzali et al., 2015). The study enables an assumption that *Sb*USP may be involved in ROS scavenging activity.

The degree of injury caused by stress in a plant is commonly measured by the physiological status of the plant. It was generally studied by measuring RWC, EL, MSI, OP, proline, and MDA content under stress condition. Ectopic expression of the *SbUSP* gene maintains the physiology of plants and a significant (*p* < 0.05) increase in relative water content, membrane stability, osmotic potential, and proline content was observed compared to control plants (WT and VC) and control condition under salinity and osmotic stresses (**Figures 7** and **8**). Lipid peroxidation and electrolyte leakage are common stress markers, which decreased in transgenic lines, overexpressing *SbUSP* gene in the present study. Malondialdehyde is the decomposition product of polyunsaturated fatty acids of biomembranes, showed greater accumulation under salt stress, the degree of accumulation of MDA was higher in Guazuncho than in Pora, indicating a high rate of lipid peroxidation in Guazuncho due to salt stress (Meloni et al., 2003). It has been demonstrated that electrolyte leakage measurement may be correlated with several physiological and biochemical parameters conditioning the plant responses to environmental conditions, such as spectral reflectance, antioxidative enzyme synthesis, membrane acyl lipid concentrations, water use efficiency, transverse relaxation time of leaf water, stomatal resistance, osmotic potential, and leaf rolling index (Bajji et al., 2002).

Lipid peroxidation (measured by MDA content) and percent electrolyte leakage were decreased significantly in transgenic lines under stress condition. Overexpression of *SpUSP* induced stomatal closure and reduced water loss in tomato under osmotic stress and thus provides drought tolerance (Loukehaich et al., 2012). Salt and drought tolerant or resistant plant including halophytes are attributed by their capacity to adjust tissue water potential to a level that is more negative than that of the soil water potential of the habitat in which they are growing (Khan et al., 2000). In the present study, transgenic lines showed an improved osmotic potential and exhibiting endurance to the plants under stress condition. The stress tolerance of plants exposed to salinity was correlated with more negative solute potential (that is a greater concentration of total osmolytes), which permitted better water retention by desiccating plants, and with a larger ratio of K^+^ to Na^+^ (Pardo and Quintero, 2002).

Osmo-protectants are cellular stress markers, which accumulate to maintain the osmotic balance across the membrane under osmotic stress. Proline, total sugar, reducing sugars, polyphenols are considered as important biochemical markers to determine the plant response under oxidative condition. The *SbUSP* gene overexpressing transgenic plants accumulated a high level of proline, reducing sugar, total sugar, free amino acids, and polyphenol (**Figures 7** and **9**). In plants, polyphenol synthesis and/or accumulation is generally stimulated in response to biotic or abiotic stresses and polyphenolic compounds indeed participate in the defense against reactive oxygen species (Ksouri et al., 2007). There were a number of reports on transgenic plants, overexpressing abiotic stress responsive gene(s) cloned from *S. brachiata*, in which osmo-protectants increase under varying salt and osmotic stress (Jha et al., 2011; Joshi et al., 2012; Chaturvedi et al., 2014; Singh et al., 2014a,b; Patel et al., 2015; Tiwari et al., 2015). Although mechanisms involved in sugar signal transduction and sugar gene regulation in higher plants are entirely not clarified yet, important progresses have been made to obtain their understanding, principally, about signals that trigger these processes and how the regulation of photosynthetic carbon metabolism interacts with other processes during stress conditions (Rosa et al., 2009).

The *SbUSP* overexpressing transgenic lines showed high K^+^ content and K^+^/Na^+^ ratio and lower accumulation of Na^+^ (Supplementary Figure S4). Previously, it was observed that salt responsive genes, such as *SbNHX1*, *SbpAPX1*, *SbASR-1* cloned from *S. brachiata* unveiled oxidative endurance to the model plant tobacco and crops, like jatropha, castor, and peanut (Jha et al., 2011; Joshi et al., 2012; Chaturvedi et al., 2014; Tiwari et al., 2014, 2015; Singh et al., 2014a,b; Patel et al., 2015). The present study unveiled the role of *SbUSP* gene in ROS scavenging mechanism directly as a non-enzymatic antioxidant or indirectly by enhancing the expression of antioxidative enzymes. To confirm its indirect role, transcript expression of *NtSOD*, *NtCAT*, and *NtAPX* genes were performed in transgenic lines and control plants subjected to control or stress (salinity and osmotic) condition (**Figure 10**).

The transcript of *NtSOD*, *NtCAT*, and *NtAPX* was induced by NaCl, desiccation and phytohormones, like ABA and SA in *SbUSP* overexpressing transgenic lines. Previously, the *SpUSP* transcript accumulated several folds under salt, drought, and ABA stress in tomato (Loukehaich et al., 2012). Similarly, a significant expression response (about eightfold) was observed for *SbUSP* under salt stress followed by drought, heat, and cold stress (Udawat et al., 2014). A high level of *GUSP* gene expression was detected in leaves, roots and stems of *Gossypium arboreum* under water stress (Maqbool et al., 2009). Li et al. (2010) reported putative barley (Steptoe) *USP* gene exhibited different expression at different time points analyzed under salt stress. Most of the drought responsive genes, studied till date, are induced by ABA and dehydration triggers the production of ABA, which in turn induces various genes (Zahur et al., 2009). Functional characterization of genes encoding ABA responsive *cis*- and *trans*-acting factors will provide useful insight to understand the ABA dependent signal transduction pathway. The ABA-responsive element (ABRE) was detected in the promoter of *SbUSP* gene. Nevertheless, a detailed involvement of ABA and SA in *SbUSP* regulation is further required to be explored. Even though, nearly 10% of the protein coding genes in *Arabidopsis* are likely to be regulated by ABA and these findings suggests that ABA-mediated gene expression plays a versatile and pivotal role in plants (Fujita et al., 2011). Although, heat stress down regulated *NtSOD*, *NtAPX*, and *NtCAT* transcript in *SbUSP* transgenic lines, in agreement to this, Kvint et al. (2003) reported that extreme temperature repressed the *USP* gene as σ^70^ gets inactivated at high temperature. In the coastal region the sea water is swamped twice in a month and remaining times the soil is almost dry, and the temperature is high.

Based on results of the study, it is hypothesized that abiotic stress responsive membrane bound cytosolic *Sb*USP protein may activate some downstream gene(s) (yet to be characterized; **Figure 14**), which lead to the accumulation of osmoprotectants to alleviate oxidative stress by scavenging ROS and thus provide salt or osmotic endurance by modulating physiological processes of the plant.

## Conclusion

Results ascertain that overexpression of the *SbUSP* gene evidently enhances salt and osmotic tolerance by maintaining physiology of the plant. Functional characterization of an autophosphorylating USP was performed and it is expected that *SbUSP* may act as “molecular knob” by interacting with components of cellular signal transduction pathway (probably MAP Kinases). The expression data in this study designate that *SbUSP* act in ABA dependent manner and affects regulation of genes responsible for ROS quenching. Although additional studies are required to establish the exact mechanism of action of the *USP* gene in the plant under abiotic stress.

## Author Contributions

Conceived and designed the experiments: AM and BJ. Performed the experiments: PU, RJ, and DS. Analyzed the data: PU and AM. Wrote the paper: PU and AM.

## Conflict of Interest Statement

The authors declare that the research was conducted in the absence of any commercial or financial relationships that could be construed as a potential conflict of interest.
