# Supplementary material for: Overexpression of a Cytosolic Abiotic Stress Responsive Universal Stress Protein (SbUSP) Mitigates Salt and Osmotic Stress in Transgenic Tobacco Plants
Source: Front Plant Sci. 2016 Apr 21;7:518. doi: 10.3389/fpls.2016.00518 (PMC4838607; doi:10.3389/fpls.2016.00518)

Supplementary Material:

Overexpression of a cytosolic abiotic stress responsive universal stress protein (SbUSP) mitigates salt and osmotic stress in transgenic tobacco plants

Pushpika Udawat, Rajesh Kumar Jha, Dinkar Sinha, Avinash Mishra* and Bhavanath Jha*
*Corresponding authors: avinash@csmcri.org (AM) and bjha@csmcri.org (BJ)

Figure S1: Molecular confirmation of transgenic tobacco plants. PCR amplification of (A) *uidA* (gus) and (B) *SbUSP* gene in T_1 transgenic lines. M: Marker (ladder), PC: Positive control, NC: Negative control, and L1-L17: Transgenic lines

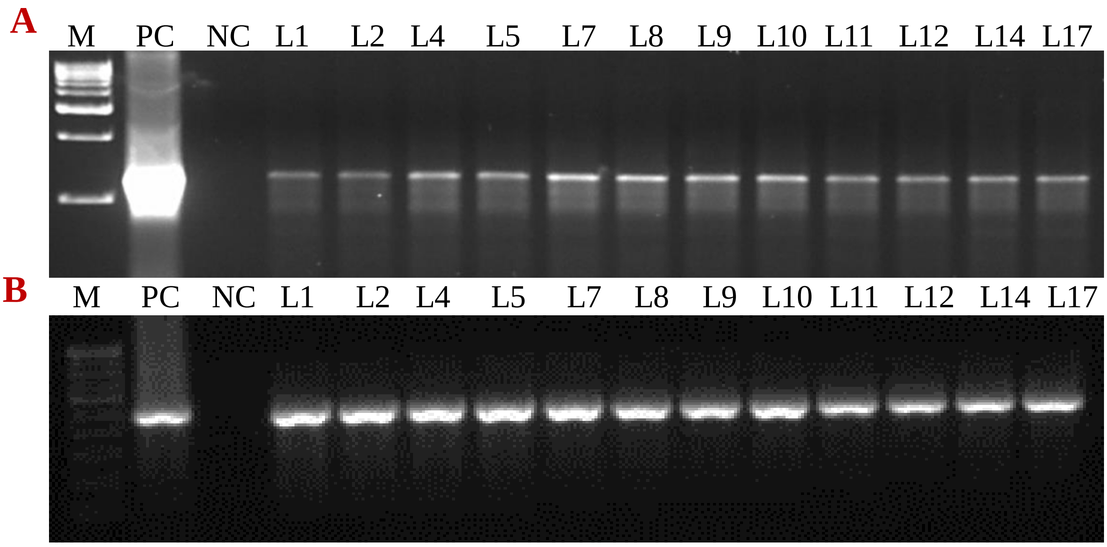

Figure S2: Molecular confirmation and overexpression analysis of transgenic tobacco plants. (A) Southern hybridization was performed to determine the transgene copy number. Overexpression of *SbUSP* gene was analyzed by Semi-quantitative reverse transcriptase PCR of transgenic lines along with control plants using (B) *SbUSP* (B) and (C) *β*-tubulin specific primers. (D) Quantitative Real Time PCR (qRT-PCR) was also performed to study the relative fold expression of *SbUSP* among transgenic lines under salinity stress. PC: Positive control, VC: Vector control, and L4-L17: Transgenic lines

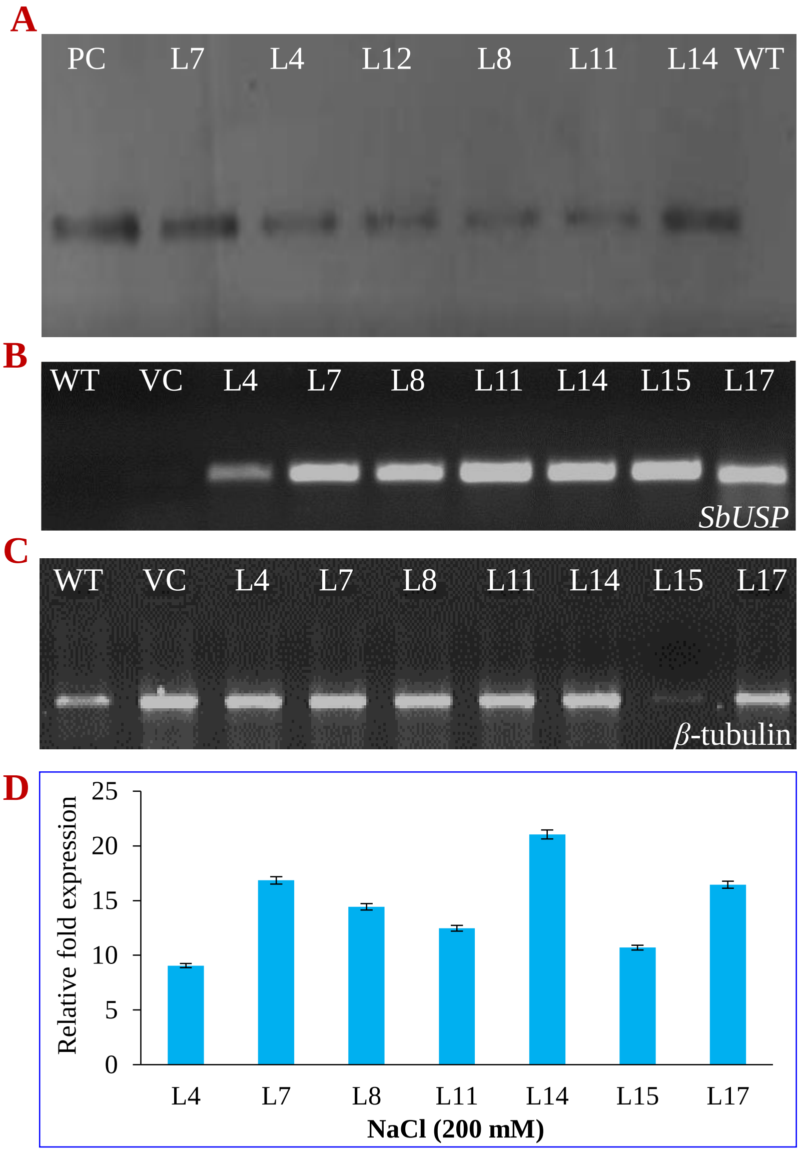

Figure S3: Quenching of superoxide radicals in transgenic lines measured by XTT assay. Bars represent means ± SE and values with different letters are significant at *P<0.05*.

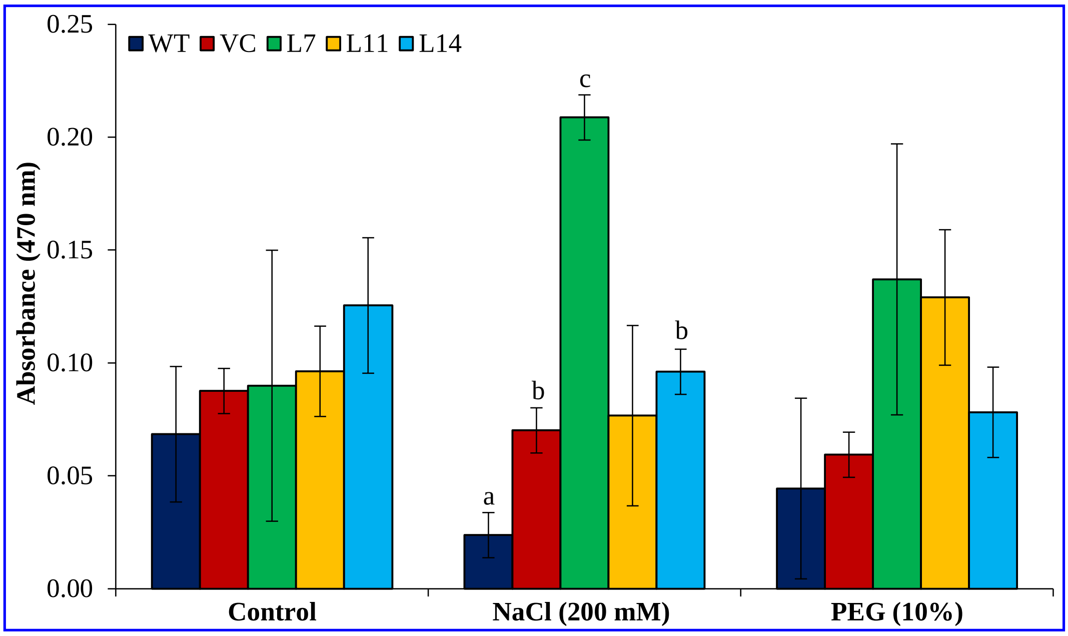

Figure S4: ICP analysis showing Na^+ and K^+ (A) and K^+/Na^+ ratio (B) homeostasis in transgenic lines. Bars represent means ± SE.

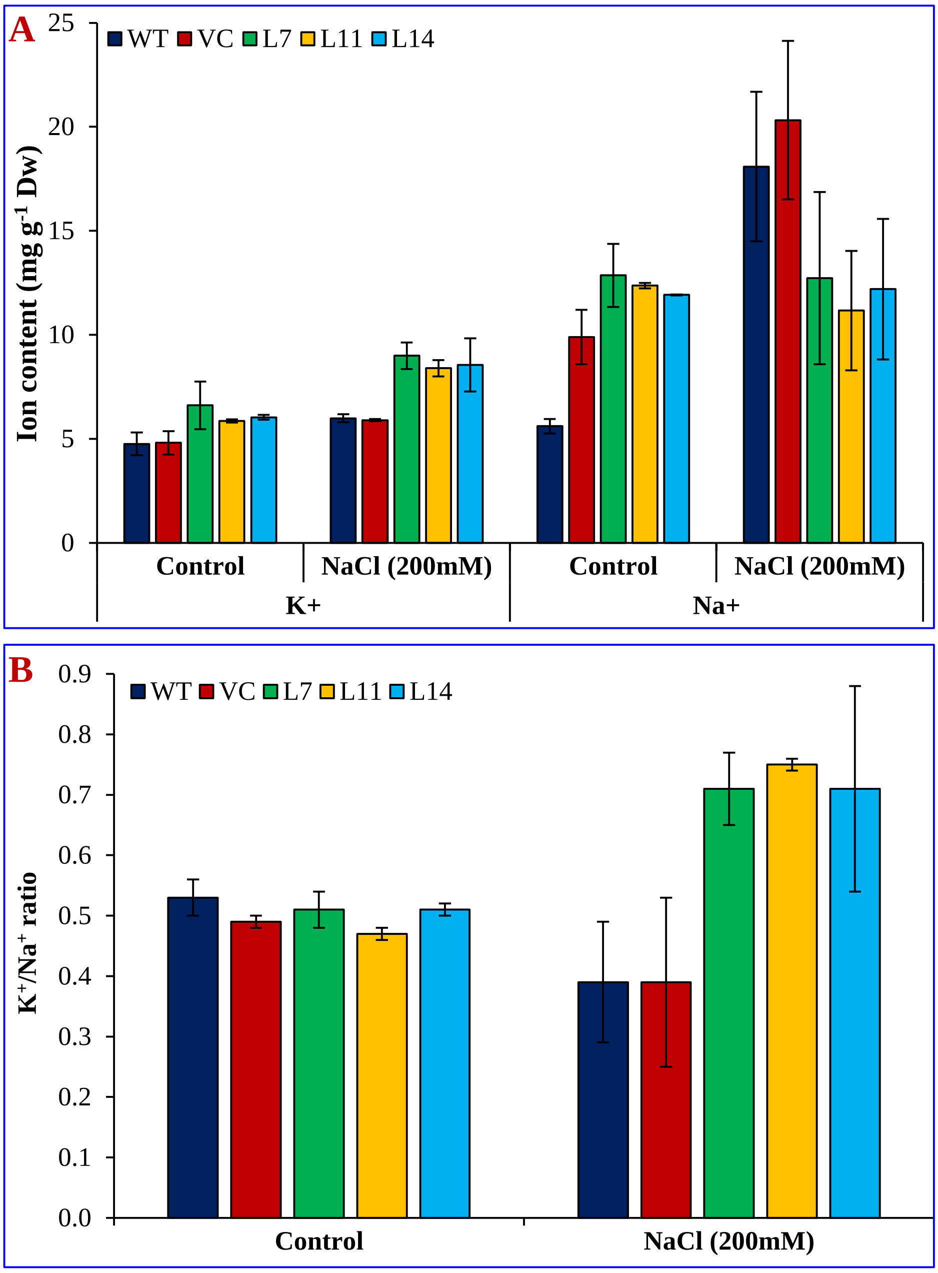

Figure S5: Heat map of differential expression of antioxidative genes under different abiotic stress condition.

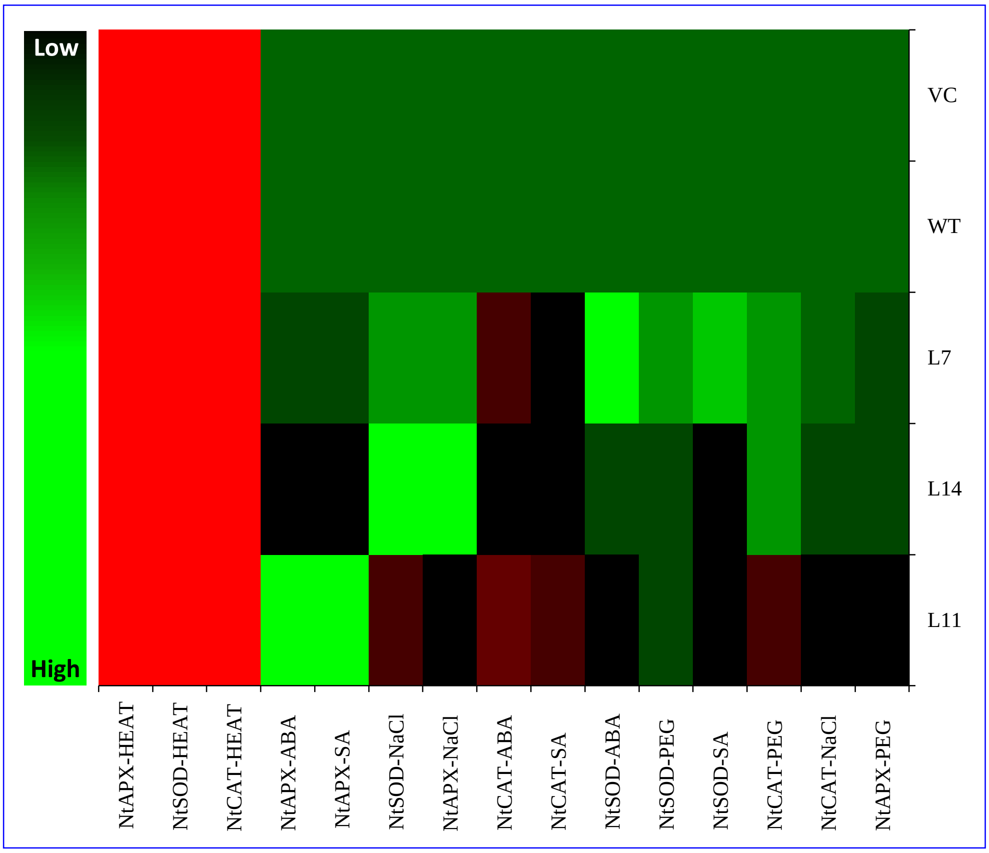

Supplement: Supplementary file 1 [file Presentation_1.PDF]
